# The impact of varus angulation on proximal fractures of the ulna

**DOI:** 10.1186/s12891-018-2012-z

**Published:** 2018-04-04

**Authors:** Xuchao Shi, Tianlong Pan, Dengying Wu, Rong Chen, Zeng Lin, Jun Pan

**Affiliations:** Department of Orthopaedics Surgery, The Second Affiliated Hospital and Yuying Children’s Hospital of Wenzhou Medical University, NO.109, Xue Yuan West Road, Wenzhou, 325027 Zhejiang Province China

**Keywords:** Varus angulation, Proximal fractures of the ulna, Malunion

## Abstract

**Background:**

We studied anteromedial varus angulation (VA) in the proximal third of the ulna. The importance of restoration of the anatomical orientation of the ulnar after a proximal fracture is unclear. The purpose of this study was to evaluate the impact of minimal proximal ulna malunion on elbow function after a proximal ulna fracture.

**Methods:**

We reviewed the follow-up of 60 patients who had undergone open reduction with internal fixation (ORIF) of a proximal fracture of the ulna. Patients were divided into two groups, defined as either more or less than 5° of the difference between the VA of the fractured and contralateral ulna. The range of motion(ROM)of elbow flexion, extension and forearm rotation on both sides, Mayo Elbow Performance Score (MEPS) and Visual Analogue Scale (VAS) were measured.

**Results:**

The average postoperative time was 3.1 years (1–5 years). Mean VA of the fractured arm was different from the normal side (7.8 ± 3.0 vs 12.7 ± 3.0). Compared to the unfractured arm there was a loss in mean elbow flexion (14.2 ± 4.9 vs 18.0 ± 5.9), extension ROM (7.1 ± 2.5 vs 9.3 ± 1.9, *p* < 0.05) and forearm rotation ROM (15.6 ± 8.6 vs 21.8 ± 9.5) that were statistically significant (*p* < 0.05). There were no statistically significant differences in the MEPS and VAS score results between the two groups (*p* > 0.05).

**Conclusions:**

The function of the elbow and forearm was restricted after VA malunion in the proximal ulna, but the quality of life of these patients had not been significantly affected. We suggest that orthopedic surgeons should assess whether the specialized structures of the proximal ulna are damaged or not before surgery. If the anatomy of the fractured bone cannot be restored through manipulation of the connected end directly, it is better to image the anatomical structure of the healthy side from using an elbow X-ray before surgery, and then reset using a pre-shaped plate to prevent malunion.

## Background

Fracture of the proximal ulnar is quite common because the elbow does not have much protection from muscle and soft tissue. If the fracture is displaced, it can be fixed using dorsal plates, tension band wiring or intramedullary screws [1–4]. For comminuted fractures, it is difficult to restore the original anatomy through splicing the fracture, so the shape of the plate largely determines the shape of the ulna after reduction. Plates for the proximal ulna are usually “anatomically pre-shaped”, but the individual anatomy of the proximal ulna can vary considerably, and so it is difficult to achieve a true anatomical reduction in every case. It is important to understand the anatomical structure of the proximal ulna and its relationship with the elbow joint after injury. Proximal ulnar morphology can influence the ROM of the elbow [5] and also fracture fixation [6, 7]. Elbow function and ROM are greater after restoration of the original anatomy of the elbow. The unique bone structures in the proximal ulna present special difficulties in the reduction of an elbow joint, fracture fixation and arthroplasty. In the existing literature, the anatomy of the adult and adolescent ulna have been defined [8–13], however, there are few reports regarding the motion of the elbow and quality of life of the patient when these particular bone structures have been disrupted or deformed.

The purpose of this retrospective study is to compare the recovery of forearm function following surgery by measuring postoperative ROM, function, pain and reoperation rates. Thus, by increasing the importance of VA to the surgeon, it may be considered more carefully following proximal ulnar fracture and so avoid malunion.

## Methods

### Patient selection

After approval by the Second Affiliated Hospital of Wenzhou Medical University, we identified all patients that were over 18 years of age that had unstable proximal ulnar fractures and had undergone fracture reduction with internal fixation between 2012 and 2016. The exclusion criteria were nonoperative treatment of fractures or dislocations associated with other parts (such as ulna and radius fractures, dislocation of the capitulum radii, etc.). Eighty patients were initially enrolled, seven of whom were unable to be contacted and 13 patients who refused to participate in the follow-up for personal reasons. Therefore, the 60 remaining patients that were contacted agreed to participate in the study. The study cohort consisted of 32 males and 28 females with a mean and standard deviation age of 46.6 and 12.7 years, respectively. For volunteers who received an invitation and gave informed consent, their personal data such as age, body mass index (BMI), if ulnar fracture occurred in dominant side(OIDS), smoking and drinking history and secondary surgery were collected.

### Study design

All volunteers were individually examined. Firstly, we undertook an X-ray measurement of the anteroposterior position of the bilateral elbow joint. All X-rays were stored using Picture Archiving and Communication System (PACS). Two trauma orthopedic surgeons independently measured each radiograph to determine interobserver reliability. All radiographs were randomly arranged to reduce observer recall bias. All patients underwent bilateral VA measurements because studies have shown that there is no significant difference in bilateral VA in the same individual [14]. The difference in VA before and after fracture could be inferred by comparing the difference in VA between the two sides, so as to determine whether the fractured forearm exhibited deformity or not. At the same time, we measured the ROM of elbow flexion and extension and forearm rotation on both sides, to compare the effect of postoperative VA healing on forearm function. These measurements were performed by the same person so as to reduce measurement error. All volunteers were invited to complete the MEPS [15] and VAS questionnaires [16]. MEPS is widely used in the evaluation of elbow fractures. The total score is 100 points, including 45 points of pain, 20 points of exercise function, 10 points of stability and 25 points of daily activities. These assessments were collated and analyzed to determine the effect of malunion on the limb of the patient. Patients were separated into two groups according to whether they exhibited VA malreduction or not, defined as more than a 5° difference between the ulna of the fractured and contralateral side.

### Statistical analysis

SPSS 22.0 (IBM Corporation, Armonk, NY, USA) was used for statistical analysis. In addition to descriptive statistics, unpaired student’s *t*-test and Fisher’s exact test were used to compare the demographic characteristics of both groups. The loss of ROM of elbow flexion, extension and forearm rotation, and MEPS and VAS score results were compared using a student’s t test. Patients were included in a Pearson correlation analysis. *P* values of 0.05 or less were considered significant.

Interobserver and intraobserver reliability were quantified using the intraclass correlation coefficient (ICC). Reliability was scored based on the criteria defined by Altman (Very good: 0.81 to 1.00; Good: 0.61 to 0.80; Moderate: 0.41 to 0.60; Fair: 0.21 to 0.40; and Poor: ≤0.20).

## Results

### Baseline characteristics for the patients

Baseline characteristics for the patients are shown in Table 1. A total of 60 volunteers participated in the study, of which 32 were males and 28 females, with a mean age of 46.6 ± 12.7. Of these, 46 patients had a dominant right hand and 14 were left hand dominant. Patients had undergone their first ORIF on average 3.1 years previously. All patients had been fixed with an ulna plate. Fifty one patients had undergone surgery a second time, all of whom had had the internal fixation surgically removed. No postoperative infection occurred and debridement was performed on 2 occasions.

**Table 1:** Demographics

	Total cohort	Group VA restored	Group VA not restored
Age (year)	46.6 ± 12.7	47.1 ± 12.6	45.8 ± 12.9
BMI	22.8 ± 3.7	22.6 ± 3.7	23.2 ± 3.7
Male	32	22	10
Female	28	18	10
Smoking	20	9	11
Alcohol consumption	29	12	17
OIDS	41	22	19
NOIDS	19	11	8
secondary surgery	51	33	18

*BMI* body mass index, *OIDS* Ulnar fracture occurred in dominant side, *NOIDS* Ulnar fracture not occurred in dominant side

### The difference in bilateral VAs

**Fig. 1:**
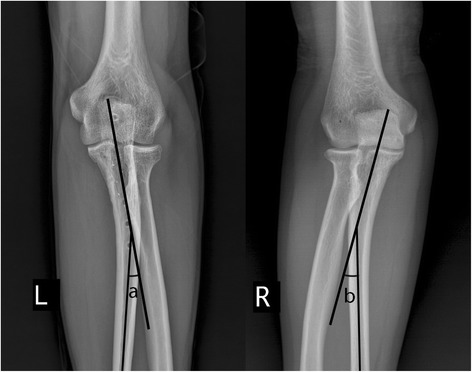
Comparison of VA measurements on both sides of the same patient

The difference in bilateral VAs can be seen in Fig. 1, where the healthy proximal ulna VA of the volunteers was 12.7 ± 3.0° as measured from X-rays, while the mean contralateral VA was 7.8 ± 3.0°, a statistically significant difference (*p* < 0.05). This indicates that the difference in preoperative and postoperative VA in volunteers was statistically significant. As we can see from Table 2, interobserver reliability was “very good” for VA.

**Table 2:** ICCs for Interobserver and Intraobserver Reliability

Variables	Interobserver	Intraobserver
VA (deg.)	0.82	0.87

*ICC* Intraclass correlation coefficient, *VA* Varus angulation

### Comparison of function of elbow joint

Data in Table 3 indicates that injury resulted in loss of maximal flexion (14.2 ± 4.9° vs 18.0 ± 5.9°), maximal extension (7.1 ± 2.5° vs 9.3 ± 1.9°) and total elbow flexion and extension (21.3 ± 6.4° vs 27.3 ± 6.0°). All three measurements were statistically significant (*p* < 0.05). Rotational ROM in the repaired compared to uninjured forearm was 15.6 ± 8.6° vs 21.8 ± 9.5°, also a statistically significant difference (*p* < 0.05).

**Table 3:** Outcomes of the elbow joint

	Group VA restored	Group VA not restored	*P* value
Loss of flexion	14.2 ± 4.9	18.0 ± 5.9	0.011
Loss of extension	7.1 ± 2.5	9.3 ± 1.9	0.001
Loss of total elbow ROM	21.3 ± 6.4	27.3 ± 6.0	0.001
Loss of forearm rotational ROM	15.6 ± 8.6	21.8 ± 9.5	0.012
MEPS	91.7 ± 4.6	91.4 ± 4.3	0.836
VAS	0.5 ± 0.9	0.6 ± 0.8	0.710

*VA* Varus angulation, *ROM* Range of motion, *MEPS* Mayo Elbow Performance Score, *VAS* Visual Analogue Scale

### Correlation between deformity degree and outcomes of the elbow joint

As we can see from Table 4, there was no statistically significant difference in MEPS and pain scale. Loss of elbow flexion, extension and forearm rotation exhibited a strong positive correlation with VA.

**Table 4:** Correlation between deformity degree and outcomes of the elbow joint

	Loss of flexion	Loss of extension	Loss of total elbow ROM	Loss of forearm rotational ROM	MEPS	VAS
Deformity degree (deg.)	0.456	0.529	0.565	0.432	−0.014	0.093

*ROM* Range of motion, *MEPS* Mayo Elbow Performance Score, *VAS* Visual Analogue Scale

## Discussion

Displaced comminuted fractures involving the proximal third of the ulna are mainly treated using a dorsal plate. When the anatomical contour of the dorsal plate is considered, individual differences in the proximal ulna must be taken into account. Its anatomy, especially the posterior margin, has clinical significance in this surgical procedure. However, the ulna is generally considered straight rather than curved, even when analyzed using three-dimensional geometry [14, 17].

The many specialized structures and curves in the proximal ulna describe VA in many studies [8, 10, 12–14, 18]. Grechenig et al. [10] defined VA as the "anterior medial angle of one third of the proximal ulna". They measured the anatomical structure of the ulna so as to assess the effect of the plate on fixation of the proximal ulnar fracture, in which mean VA was 17.5° (11–23°). Windisch et al. [12] measured the elbow joints of 74 cadavers, finding a mean VA of 17.7° (11–28°). Totlis et al. [14] measured 200 ulna, Puchwein et al. [18] studied 40 and Beser et al. [8] measured 50 ulna, finding a mean VA of 8.48° (2.1–15.7°), 14.3 ± 3.6° and 9.3 ± 2.2° (4.0–15.0°), respectively. There are two reasons for the large differences in the reported measurements. Firstly, different researchers chose different reference points: Beser et al. [8] and Totlis et al. chose the intermediate bone shaft (ulnar midshaft axis), while three other studies chose the posterior margin of the ulna. Secondly, variability in VA was quite large, VA of different sexes in the same study were also statistically different [14].

Because there is a great difference in VA within the population, restoration of proximal ulna fractures requires attention to individual differences, in order to correctly design the internal fixation plate. Since the lateral angle of the VA varies considerably, it may not be possible to achieve a true anatomical reduction if the fracture is adjusted to fit the shape of an "anatomically prefabricated plate". If it eventually leads to malunion, it may affect the function of the forearm. Therefore, understanding the effect of non-anatomical reduction of VA on the function of the affected limb requires further confirmation.

In our study, we could not measure the posterior border of the ulna because of the use of X-ray film instead of dissecting cadaveric bone, so the ulna midshaft axis was measured instead. The subjective basis for these measurements is large, that is, they require the examiner to subjectively create 2 straight lines in 2 different bony segments. The reliability between observers was very high in our study. The VA decreased by more than 5 degrees in 38 patients. We measured elbow flexion, stretching, total ROM and forearm rotation ROM reduction in these patients, and demonstrated that restoration of VA anatomy is critical for the recovery of ROM in the forearm. The anatomical plates were not in accordance with the specific structure of the proximal ulna, because of individual differences. Therefore, we suggest that X-rays of the two proximal ulna should be performed at the same time after ORIF of the fracture. Pre-shaped anatomical plates of the proximal ulna matching the VA on the healthy side, especially for comminuted fractures which cannot be reset by fracture block splicing, are particularly important, as preoperative preparation often determines the quality of life of the patients after surgery.

However, there was no statistical difference between the MEPS of the two groups so that while it can be seen that the ROM of the forearm was limited, it presented no obvious obstacle to everyday activities. The level of pain between the two groups was not statistically significant, as a long time had elapsed since the surgery. Most patients had had the internal fixation removed and their scar had stabilized. Loss of elbow flexion, extension and forearm rotation had a strong positive correlation with VA, meaning that as the deformity angle of the VA increased the ROM of the elbow and forearm decreased. Thus, we recommend that orthopedic surgeons consider the series of specialized anatomical structures of the proximal region of the ulna at the time of surgery to prevent malunion of the fracture, thereby affecting function of the patient’s forearm.

However, our research has some limitations. First of all, measurement of VA and answering the questionnaire is subjective, which may impact the results of the study. Secondly, the sample size of our study was not large enough to reach firm conclusions. Finally, the retrospective nature of the present study makes it impossible to evaluate the impact of soft tissue damage, rehabilitation programs or immobilization time on the final outcome of function.

## Conclusions

Elbow and forearm function can become limited following malunion in a proximal fracture of the ulna, but quality of life of the patients was not significantly affected. In view of this result, we suggest that orthopedic surgeons assess if the specialized structures of proximal ulna have been damaged prior to surgery. If the anatomy of the fractured bone cannot be directly restored, it is better to measure the anatomical structures of the healthy side using X-rays before the operation, then reset using a pre-shaped plate to prevent malunion.

## Abbreviations

BMI: Body mass index
ICC: Intraclass correlation coefficient
MEPS: Mayo Elbow Performance Score
OIDS: Ulnar fracture occurred in dominant side
ORIF: Open reduction with internal fixation
PACS: Picture archiving and communication system
ROM: Range of motion
VA: Varus angulation
VAS: Visual Analogue Scale
